# *Stachys* Species: Comparative Evaluation of Phenolic Profile and Antimicrobial and Antioxidant Potential

**DOI:** 10.3390/antibiotics12111644

**Published:** 2023-11-20

**Authors:** Daniela Benedec, Ilioara Oniga, Daniela Hanganu, Brîndușa Tiperciuc, Adriana Nistor, Ana-Maria Vlase, Laurian Vlase, Cristina Pușcaș, Mihaela Duma, Cristian Cezar Login, Mihaela Niculae, Radu Silaghi-Dumitrescu

**Affiliations:** 1 Department of Pharmacognosy, “Iuliu Hațieganu” University of Medicine and Pharmacy, 12 I. Creanga Street, 400010 Cluj-Napoca, Romania; dbenedec@umfcluj.ro (D.B.); ioniga@umfcluj.ro (I.O.); adriana.nistor@elearn.umfcluj.ro (A.N.); 2 Department of Pharmaceutical Chemistry, “Iuliu Hațieganu” University of Medicine and Pharmacy, 41 V. Babeş Street, 400012 Cluj-Napoca, Romania; btiperciuc@umfcluj.ro; 3 Department of Pharmaceutical Botany, “Iuliu Hațieganu” University of Medicine and Pharmacy, 12 I. Creanga Street, 400010 Cluj-Napoca, Romania; gheldiu.ana@umfcluj.ro; 4 Department of Pharmaceutical Technology and Biopharmacy, “Iuliu Hațieganu” University of Medicine and Pharmacy, 41 V. Babeş Street, 400012 Cluj-Napoca, Romania; laurian.vlase@umfcluj.ro; 5 Department of Chemistry and Chemical Engineering, Babes-Bolyai University, 11 A. Janos Street, 400028 Cluj-Napoca, Romania; cbischin@chem.ubbcluj.ro (C.P.); radu.silaghi@ubbcluj.ro (R.S.-D.); 6 State Animal Health and Safety Veterinary Laboratory, 1 Piata Marasti Street, 400609 Cluj-Napoca, Romania; 7 Department of Physiology, “Iuliu Hațieganu” University of Medicine and Pharmacy, 1 Clinicilor Street, 400006 Cluj-Napoca, Romania; cezar.login@umfcluj.ro; 8 Department of Clinical Sciences, University of Agricultural Sciences and Veterinary Medicine Cluj-Napoca, 400372 Cluj-Napoca, Romania; mihaela.niculae@usamvcluj.ro

**Keywords:** *Stachys*, polyphenols, cytochrome *c*, antimicrobial, antioxidant

## Abstract

This study aimed to investigate the polyphenolic composition and antioxidant and antimicrobial potential of six Romanian Stachys species: *S. officinalis*, *S. germanica*, *S. byzantina*, *S. sylvatica*, *S. palustris*, and *S. recta*. The LC-MS/MS method was used to analyze the polyphenolic profile, while the phenolic contents were spectrophotometrically determined. The antioxidant activity was evaluated using the following methods: DPPH, FRAP, nitrite-induced autooxidation of hemoglobin, inhibition of cytochrome *c*-catalyzed lipid peroxidation, and electron paramagnetic resonance spectroscopy. The in vitro antimicrobial properties were assessed using agar-well diffusion, broth microdilution, and antibiofilm assays. Fifteen polyphenols were identified using LC-MS and chlorogenic acid was the major component in all the samples (1131.8–6761.4 μg/g). *S. germanica*, *S. palustris*, and *S. byzantina* extracts each displayed an intense antiradical action in relation to high contents of TPC (6.40 mg GAE/mL), flavonoids (3.90 mg RE/mL), and caffeic acid derivatives (0.89 mg CAE/mL). In vitro antimicrobial and antibiofilm properties were exhibited towards *Candida albicans*, Gram-positive and Gram-negative strains, with the most intense efficacy recorded for *S. germanica* and *S. byzantina* when tested against *S. aureus*. These results highlighted Stachys extracts as rich sources of bioactive compounds with promising antioxidant and antimicrobial efficacies and important perspectives for developing phytopharmaceuticals.

## 1. Introduction

As a member of the Lamiaceae family, the *Stachys* L. genus (wound plants) has garnered scientific interest regarding its chemical composition and therapeutic properties. In fact, numerous species of this genus have been used empirically in different regions of the world for their medicinal and economic potential since ancient times [1,2]. 

A recently published paper reviewed the existing information on various traditional uses of 25 species and 6 subspecies of *Stachys* genus, conditioned mostly as herbal tea, infusion, and decoction, but also as extracts (oily and alcoholic) and edible food (tubers) [2]. This study also described the phytochemistry and pharmacological profile of distinct *Stachys* sp. originating from the Mediterranean region, Asia, America, and southern Africa. 

Overall, a complex chemical composition including secondary metabolites such as flavonoids (baicalin, apigenin, isoscutellarein 7-*O*-β-D-glucopyranoside), phenolcarboxylic acids (caffeic, rosmarinic, chlorogenic acids), tannins, phenylethanoid glucosides (acteoside, martinosid), lignans, iridoids, diterpenes (kauran, clerodane), and triterpenes; essential oils characterize *Stachys* genus [1,2].

Moreover, several *Stachys* species have been studied in vitro and in vivo to scientifically justify their traditional use. The pharmacological assessment demonstrated important therapeutic properties, namely the following: antioxidant (*S. recta* subsp. *recta*, *S. palustris*, *S. byzantina*, *S. sylvatica*), anti-inflammatory (*S. recta*, *S. germanica*, *S. officinalis*, *S. alpina*), analgesic, anxiolytic, antidepressant, antiproliferative (*S. lavandulifolia*), antibacterial (*S. byzantina*, *S. officinalis*, *S. sylvatica*), renoprotective, and hepatoprotective (*S. pilifera*) [1,2]. 

Although *Stachys* sp. are spreading in the Romanian flora [3,4], only a few references are available [5,6,7,8]. In this regard, common and Latin names, botanical descriptions, and geographical distribution are presented for 14 species of *Stachys*, all perennial herbaceous plants, in the *Flora of Romania* [3,4]. Among these, *Stachys officinalis* (L.) Trevis. (sin. *Betonica officinalis* L.) is the only *Stachys* species documented by Romanian traditional medicine. Phytopreparations obtained from its leaves and aerial parts are mentioned for tonic, antipyretic, analgesic, and cicatrizing efficacy, and have been traditionally recommended for various disorders such as rhinitis, headache, hemoptysis, chest pain, wounds, and varicose ulcers [5,6]. Nowadays, most of the empirical therapeutic applications involve *S. officinalis* tea in atonic dyspepsia and liver disorders based on antispastic, astringent, carminative, cholagogue, digestive stimulant, emmenagogue, anthelmintic, and diuretic properties. The tea is also listed for anxiolytic and sedative effects, being able to reduce anxiety and irritability [6]. Regarding other *Stachys* species, *S. byzantine* is a very adaptable and decorative plant with brightly colored flowers and is commonly cultivated for ornamental purposes [3].

Previous studies on Romanian *Stachys* extracts addressed the following species: *S. officinalis* [8], *S. byzantina*, and *S. sylvatica* [7,8]. 

The current study objective was to evaluate the polyphenolic composition, antioxidant, and in vitro antimicrobial potential of *Stachys* species frequently found in the Romania flora: *S. officinalis*, *S. germanica*, *S. byzantina*, *S. sylvatica*, *S. palustris*, and *S. recta*. These data are required to complete these species’ pharmacognostic profiles aiming to ensure a well-deserved place in phytotherapy. Thus, additional chemical and pharmacotherapeutic data are needed to reveal the most promising *Stachys* species for medicinal use.

## 2. Results and Discussions

### 2.1. Chromatographic Analysis

To characterize plant extracts rich in polyphenols, the most widely used technique for the separation and individual quantification of phenolic compounds is liquid chromatography coupled with tandem mass spectrometry (LC-MS/MS) [9,10]. The amounts of compounds detected in the six *Stachys* sp.-derived extracts were expressed in μg/g dry weight (dw) of vegetal product (Table 1).

Fifteen polyphenolic compounds, including ten phenolic acids (gallic, rosmarinic, protocatehuic, gentisic, caftaric, chlorogenic, vanillic, syringic, *p*-coumaric, ferulic acids) and five flavonoids (isoquercitrin, rutin, quercitrin, luteolin, apigenin), were detected by LC-MS in all *Stachys* extracts (Table 1). 

Furthermore, all extracts presented high amounts of chlorogenic acid (1131.8–6761.4 μg/g dw). Chlorogenic acid with multiple therapeutic potentials (neuroprotective; cardio-, hepato-, gastro-, renoprotective; anticancer) is a phenolic acid widely distributed in many species of the *Stachys* genus [2,11]. The extracts richest in chlorogenic acid were from *S. germanica* (4205.2 μg/g dw), *S. sylvatica* (5552.7 μg/g dw), and *S. recta* (6761.4 μg/g dw). High concentrations of chlorogenic acid were measured in Turkish *S. germanica* [12], but this active principle was not found in *S. recta* [13]. The other two indigenous *Stachys*: *S. officinalis* (1131.8 μg/g dw) and *S. byzantina* (2012.1 μg/g dw) showed lower concentrations of chlorogenic acid compared to the other species, but higher than some published values [14]. The results obtained for the *S. officinalis* extract are similar to those previously reported by other authors [15]. 

*S. officinalis* presented higher amounts of vanilic acid (83.5 μg/g dw), syringic acid (82.7 μg/g dw), ferulic acid (11.1 μg/g dw), and apigenin (84.2 μg/g dw) than the other *Stachys* species. Bączek et al. determined ferulic acid and apigenin in Polish *S. officinalis* in higher concentrations [16] and Paun et al. have published lower values of apigenin than ours [17]. Regarding protocatechuic and *p*-coumaric acids, the *S. recta* extract was found to be the richest in these compounds, at 157.8 μg/g dw and 32.0 μg/g dw, respectively.

Caftaric acid was determined only in *S. officinalis* (4.7 μg/g dw) and gallic acid only in *S. palustris* (141.5 μg/g dw). 

The *S. officinalis* extract (82.7 μg/g dw) was the richest in syringic acid, compared to the *S. sylvatica* (8.6 μg/g dw), *S. byzantina* (7.0 μg/g dw), and *S. palustris* (4.4 μg/g dw) extracts. Four species of *Stachys* (*S. officinalis*, *S. recta*, *S. sylvatica*, *S. germanica*) contained isoquercitrin; the richest species was *S. germanica* (233.1 μg/g dw). Isoquercitrin, also identified by other authors in *S. recta* [13], develops numerous chemoprotective effects in vitro and in vivo against oxidative stress, diabetes, allergies, cancer, etc. [17]. 

Rutin and quercitrin were found in very small quantities, below the limit of detection in the extracts of *S. officinalis* (rutin,) and *S. palustris* (quercitrin).

The characterization of polyphenolic profiles of the six *Stachys* species showed differences in the presence of key compounds, but also in what concerns the concentrations of individual phenols.

Numerous medicinal species in the Lamiaceae family contain rosmarinic acid (RA), a natural polyphenolic molecule with antioxidant, antidiabetic, antimicrobial, hepatoprotective, cardioprotective, nephroprotective, anti-aging, anti-inflammatory, antiviral, and anticancer therapeutic properties [18]. Data from the literature confirm the presence of RA in other species of *Stachys*: *S. thirkei* [19], *S. tmolea* [20], *S. cretica* [21], *S. lavandulifolia*, *S. byzantina* [22], and *S. officinalis* [15,23].

In this case, we chose to use the LC-MS/MS method, published by us, to determine the RA content of the six *Stachys* species [24]. 

The ESI source mass spectrometer, in the negative mode, was set to isolate and fragment the deprotonated RA molecule with *m*/*z* = 359. The quantification of RA was performed with a tR of 2.2 min. In the range of 40–640 ng/mL, a linear calibration curve was performed (R^2^ = 0.999). The RA content values (μg/g dw) are presented in Table 1; this amount varied between 1.9–43.4 μg/g dw of vegetal product.

The *S. officinalis* extract showed a higher RA content (43.4 μg/g dw) compared to the other *Stachys* species extracts studied by us, but lower than the results published by other authors [15,16]. From the data in the literature accessed by us until now, we can say that RA was found for the first time in the extracts of *S. germanica* (7.8 μg/g dw), *S. recta* (2.6 μg/g dw), *S. palustris* (2.4 μg/g dw), and *S. sylvatica* (1.9 μg/g dw). Of the six spontaneous native species of *Stachys*, *S. officinalis* can be considered as an important source of rosmarinic acid.

The ethanolic extracts of *Stachys* sp. were examined to determine the polyphenolic TPC), flavonoid, and caffeic acid derivatives contents. The results obtained in this study were expressed as gallic acid (GA), rutin (R), and caffeic acid (CA) equivalents, respectively (Table 2). 

Medicinal plants produce polyphenols with various chemical structures, such as flavonoids, phenolic acids, with important antioxidant, cytotoxic, anti-inflammatory, antihypertensive, and antidiabetic properties [25]. The TPC contents of these six *Stachys* sp. extracts, determined using the Folin−Ciocâlteu reagent [26], are shown in Table 2. According to this analysis, *S. byzantina* extract had the highest concentration of TPC (6.40 mg GAE/mL) and the *S. palustris* and *S. germanica* extracts had similar values (5.35 mg GAE/mL and 5.98 mg GAE/mL, respectively). The other three species showed lower values, between 2.51 mg/mL and 4.00 mg GAE/mL, the *S. officinalis* extract being the lowest in TPC (2.51 mg GAE/mL). In terms of TPC, the extract of *S. byzantina* was the richest in these active compounds. The statistical analysis of these data (Table 2) showed highly significant differences between *S. byzantina* (SB) and other *Stachys* sp.: SO, SG, SS, and SR (*p* < 0.001) and significant differences between *S. byzantina* (SB) and *S. palustris* (*p* = 0.011). Our results were similar to those published by other authors for *S. byzantina* and *S. germanica* species, [27,28] while other authors described lower values than ours for *S. officinalis* [15].

The total flavonoid contents were determined using the AlCl_3_ method [29]. The highest content of flavonoids, with values between 3.14 and 3.90 mg RE/mL (*p* > 0.05), was found in ethanolic extracts of *S. palustris*, *S. germanica*, and *S. byzantina*. The other extracts of *S. officinalis*, *S. sylvatica*, and *S. recta* extracts had lower amounts of flavonoids, around 1 mg/mL (1.13, 1.18, and 1.22 mg RE/mL, respectively, in relation with *S. byzantina* (0.001 < *p* < 0.05). Comparing different studies from the literature, some authors have reported lower levels of flavonoids in *S. officinalis* [15], *S. germanica*, *S. sylvatica*, and *S. recta* [30]. Sytar et al. found higher concentrations of flavonoids in *S. byzantina* [14] compared to our results, and Nikolova recorded similar values for *S. officinalis* [30].

Caffeic acid derivatives, which are widespread in many plant species, have been extensively studied; this has revealed a wide range of potential therapeutic applications. Regarding the contents of caffeic acid derivatives for the *Stachys* sp. extracts, values ranged between 0.20–0.89 mg CAE/mL (Table 2). The highest values were obtained for the ethanolic extracts of *S. palustris* (0.72 mg CAE/mL), *S. germanica* (0.88 CAE mg/mL), and *S. byzantina* (0.89 CAE mg/mL), while the lowest were measured in the extracts of *S. officinalis* (0.20 mg CAE/mL), *S. sylvatica* (0.20 mg CAE/mL), and *S. recta* (0.23 mg CAE/mL), compared to *S. byzantina* (0.001 < *p* < 0.05). The literature provides data only for *S. germanica* caffeic acid derivatives’ content; our results varied slightly compared to those published by Mitic et al. [28].

Qualitative and quantitative evaluation of the phenolic profile indicated S. *germanica*, *S. byzantina*, and *S. palustris* as the species with the richest content in these active compounds including flavonoids and caffeic acid derivatives.

### 2.2. Antioxidant Activity of Stachys *sp.* Extracts

Bioactive compounds of the polyphenol type are important natural antioxidants due to their redox characteristics, as a result of hydroxyl groups that facilitate the elimination of free radicals and thus offer many benefits to human health [25]. In this regard, extracts of *Stachys* sp. with polyphenols, have been evaluated by several antioxidant methods: the DPPH radical scavenging method, the FRAP test (the ferric-reducing ability of plasma), nitrite-induced autooxidation of hemoglobin (NHA), inhibition of cytochrome *c*-catalyzed lipid peroxidation, and EPR spectroscopy assays (Table 3). 

The results of DPPH scavenging activity revealed that five *Stachys* extracts presented high antioxidant activity: *S. byzantina* (IC_50_ = 53.61 µg/mL), *S. germanica* (IC_50_ = 54.18 µg/mL) and *S. palustris* (IC_50_ = 54.82 µg/mL) extracts, followed by extracts of *S. sylvatica* (IC_50_ = 76.04 µg/mL) and *S. recta* (IC_50_ = 84.94 µg/mL), but lower compared to Trolox (^a^ *p* < 0.001). In contrast, the *S. officinalis* extract showed a higher value of IC_50_ (139.16 µg/mL), which indicates a lower activity. Other authors have obtained IC_50_ values higher than ours (*S. officinalis*, *S. germanica*, *S. sylvatica*, *S. byzantina*) [13,30]. Similar results were obtained for *S. officinalis* from Romania [15], as well as for the Bulgarian *S. recta* species [30].

The FRAP values for the investigated extracts were reported in Table 3, so that *S. germanica* and *S. palustris* extracts showed antioxidant activity (688.21 and 502.43 μM TE/mL), in relation to high polyphenol contents (5.77 and 5.56 mg GAE/mL), but also with the values obtained by the DPPH method (53.83 and 55.66 µg/mL). For the other four *Stachys* sp. extracts, lower values were obtained (between 218.17and 354.88 μM TE/mL) than *S. germanica* (^b^ *p* < 0.001). The NHA results on all *Stachys* extracts offer values in the same order and proportion as the FRAP results (e.g., highest results for *S. germanica* and lowest results— for *S. sylvatica*, by about three times). This excellent agreement between FRAP and NHA was not mirrored by the DPPH results. Thus, in the DPPH measurements *S. sylvatica* was far from being the weakest antioxidant, while *S. germanica* was narrowly exceeded as the strongest oxidant by *S. byzantina*—even though the two had widely different activities in the NHA assay. Interestingly, the NHA data does not correlate with either the total phenolic of flavonoid contents or with the content of any particular component (including the major ones—rosmarinic acid, chlorogenic acid, and caffeic derivatives). The NHA assay tests a nitrosative stress process, while FRAP was based on oxidative stress agents. The excellent agreement between NHA and FRAP, together with the fact that NHA is based on a physiological reaction employing a protein from blood, may be taken as argument that these two assays have a particularly important biomedical/physiological relevance. Regarding the FRAP values published by other authors, important results were obtained for *S. germanica subsp. heldreichii* [12]; the species *S. byzantina* and *S. sylvatica* showed a lower antioxidant activity [27,31,32,33].

An antioxidant assay based on the inhibition of cytochrome-catalyzed lipid autooxidation was also attempted. However, in this case the *Stachys* extracts exhibited effects that were too narrowly similar to each other—although *S. germanica* was still found at the most-active end of the range, while *S. sylvatica* was at the least-active end.

**Figure 1:**
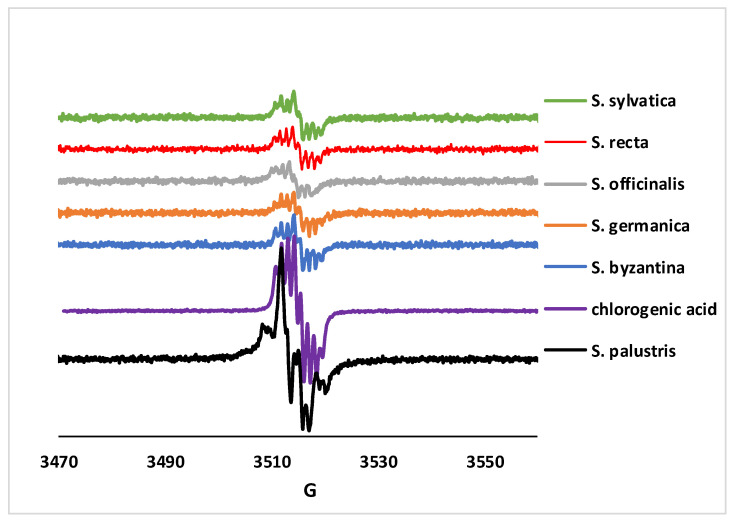
EPR spectra of the extracts treated with NaOH. Conditions: extracts—0.5%, NaOH—5 mM, and Et-OH—90%; G—magnetic field strength.

Exposure of phenolic compounds to basic pH under aerobic conditions allows for the generation of phenol-based free radicals via autooxidation processes. Such radicals can be detected by EPR spectroscopy and can be used as fingerprints for assessing the dominant components in polyphenolic-containing natural extracts. Figure 1 shows that a clear complex signal was detected for all the *Stachys* extracts, which is very similar to that of pure chlorogenic acid samples. This observation was in line with the fact chlorogenic acid was by far the most abundant polyphenolic compound in these samples, that according to the chromatographic data in Table 1; while up to a sixfold difference in chlorogenic acid concentrations is seen in Table 1, Figure 1 offers a spectra of intensities that were essentially equal among all samples. A single exception exists—namely, the *S. palustris* sample, for which the EPR spectrum was no longer identical to that of chlorogenic acid. This implies that *S. palustris* contains a unique antioxidant species, not shared with the other *Stachys* extracts. Table 1 and Table 2 reveal only one such antioxidant—namely, gallic acid, which is present only in *S. palustris*. Indeed, the shape of the *S. palustris* EPR spectrum was consistent with that of a gallate radical imposed over chlorogenic acid [34].

In the present study, it was observed that the extracts of *S. germanica*, *S. palustris*, and *S. byzantina* had the highest antiradical action, in a general relation to a high content of TPC, flavonoids, and caffeic acid derivatives. The *S. recta* and *S. sylvatica* extracts with a medium content of polyphenols also presented a high antioxidant capacity. Thus, it can be concluded from the evaluation of the phenolic content and the antioxidant activity that these species of *Stachys* can be recommended as valuable natural antioxidants for various nutritional functions and to preserve health.

### 2.3. Antimicrobial Activity of Stachys *sp.* Extracts

The use of natural herbal preparations as antimicrobial agents is a therapeutic alternative, especially in the context of the increasing bacterial resistance to antibiotics. In addition, it is known from the literature that *Stachys* species have many pharmacological activities (anti-inflammatory, antianxiety, antinephritic, antioxidant, cytotoxic) [12,27,35,36] and antimicrobial properties have been demonstrated for some species, such as *S. byzantina*, *S. guyoniana*, *S. officinalis*, *S. sylvatica*, *S. cretica*, *S. scardica*, *S. recta*, and *S. germanica*. [3,23,37,38,39,40,41]. 

#### 2.3.1. Antibacterial Activity Using the Agar-Well Diffusion Method

Using the agar-well diffusion method, we evaluated the antimicrobial susceptibility of significant bacterial isolates of the six indigenous *Stachys* species (Table 4) [42]. Our extracts showed a strong antibacterial activity, especially against *S. aureus* and *L. monocytogenes*, with areas of inhibition ranging from 14 and 20 mm, compared to gentamicin (IZD = 18 mm), used as a reference antibiotic (0.001 < *p* < 0.05). The activity on *S. aureus* was almost the same as gentamicin for *S. officinalis*, *S. sylvatica*, and *S. recta* (*p* < 0.05) and was superior for *S. germanica* and *S. byzantina* with IZD = 20 mm (*p* < 0.001). The same IZD (18 mm) was measured for the *S. palustris* extract as for the antibiotic standard (*p* = 1). All extracts showed lower activity on *L. monocytogenes* (*p* < 0.001) compared to gentamicin used as reference antibiotic. 

Regarding the antibacterial activity against Gram-negative strains, *Stachys* extracts showed limited activity against *E. coli* and *S. enteritidis*, statistically lower compared to gentamicin (*p* < 0.001). These results were consistent with those published by other authors, according to which Gram-negative bacteria display less sensitivity to the effect of *Stachys* extracts compared to Gram-positive ones [3,32,38,43]. Also, similarly to previous reports, our *Stachys* extracts showed a relatively low anti-*C. albicans* activity compared to Fluconazole (*p* < 0.001), which was used as the reference antifungal [38,44].

#### 2.3.2. Antibacterial Activity Using the Broth Microdilution Method

Moreover, the results obtained using the broth microdilution method (Table 5) showed *Stachys* extracts higher inhibitory efficacy towards Gram-positive bacteria, *S. aureus*, and *L. monocytogenes*. The lowest values of MIC were recorded for *S. germanica* and *S. byzantina*. These two extracts were found also with the lowest MBC values established against *S. aureus*, suggesting their superior bactericidal potential towards this bacterial species. All *Stachys* extracts presented in vitro inhibitory and bactericidal potential against *E. coli* and *S. enteritidis*, but only at the highest tested concentrations (Table 5). *C. albicans* growth was inhibited by all tested extracts and a two- and threefold higher fungicidal effect was noticed for MBC values than for MIC values, respectively.

#### 2.3.3. Antibiofilm Activity

The inhibitory activity of *Stachys* sp. extracts against biofilm formation is presented in Table 6.

Both the biofilm attachment (T0) and destruction of 24 h pre-formed biofilm (T24) were inhibited by certain extracts, the most intense effect (++) was still noticed against the planktonic cells. The highest efficacy was recorded for *S. germanica*, *S. byzantina*, *S. sylvatica*, and *S. palustris*-derived extracts.

According to the literature, *S. byzantina*, *S. officinale*, and *S. sylvatica* showed strong antibacterial activity, *S. aureus* being very susceptible to these extracts [3,32,38]. The antibacterial activity of the *Stachys* sp. extracts could be due to the presence of chlorogenic acid, which is found in large quantities in our extracts and according to studies can increase the permeability of the outer membrane and plasma and thus lead to the loss of the barrier function. It also depletes intracellular potential and releases cytoplasmic macromolecules, which can lead to bacterial cell death [46,47].

The results of this study pointed out that *Stachys* extracts were able to display an important in vitro antimicrobial potential against Gram-positive strains, with the extracts of *S. germanica*, *S. byzantina*, and *S. palustris* achieving very good antistaphylococcal activity. This aspect is relevant as *S. aureus* is regarded worldwide as one of the most important human bacteria, with both colonizing and opportunistic pathogen valences and elevated levels of multidrug resistance [48]. In this regard, the results indicated *S. germanica* and *S. byzantina* extracts for their superior antibiofilm properties against *S. aureus*.

## 3. Materials and Methods

### 3.1. Plant Material

The aerial parts from six *Stachys* species: *S. officinalis*—SO, *S. germanica*—SG, *S. byzantina*—SB, *S. sylvatica*—SS, *S. palustris*—SP, and *S. recta*—SR (Voucher numbers: 106-111) were collected from the spontaneous flora of Cluj County, Romania (Podeni village: 46°41′4″ N 23°1′45″ E), during the flowering period. The plant material was identified by Ph.D. A. Mocanu (“Iuliu Haţieganu” University of Medicine and Pharmacy Cluj-Napoca).

### 3.2. Chemicals 

All high-quality purity chemicals (substances and reagents) used in this study were acquired from the following companies: Sigma, St. Louis, MO, USA (chlorogenic, cafeic, *p*-coumaric acids, isoquercitrin, quercitrin, hyperoside, rutin, apigenin, myricetin, quercetin, kaempferol, fisetin); Roth, Karlsruhe, Germany (sinapic, ferulic, gallic, gentisic, rosmarinic acids, patuletin, luteolin); Dalton, Toronto, ON, Canada (caftaric, cichoric acids); Alfa-Aesar, Karlsruhe, Germany (sodium hydroxide, sodium carbonate, and iron (III) chloride); Sigma-Aldrich, Steinheim, Germany (soy lecithin, bovine hemoglobin, protocatechuic, vanillic and syringic acids, (−)-epicatechin, (+)-catechin), 6-hydroxy-2,5,7,8-tetramethylchroman-2-carboxylic acid (Trolox), 2,4,6-Tris (2-pyridyl)-s-triazine (TPTZ), sodium acetate buffer solution); Merck, Darmstadt, Germany (Arnow’s reagent, Folin−Ciocâlteu reagent, methanol, ethanol, aluminum chloride, sodium acetate, hydrochloric acid, 2,2-diphenyl-1-picrylhydrazyl; the methanol, acetonitrile, ammonium acetate, and acetic acid were all of the HPLC grade).

The Gram-positive bacteria: *Staphylococcus aureus* (ATCC 6538P) and *Listeria monocytogenes* (ATCC 13932); Gram-negative bacteria: *Escherichia coli* (ATCC 25922) and *Salmonella typhimurium* (ATCC14028); and one fungal strain, *Candida albicans* (ATCC 10231), were distributed by MicroBioLogics^®^, St. Cloud, MN, USA.

The spectrophotometric data were acquired using a Jasco V-530 UV–Vis spectrophotometer (Jasco International Co., Ltd., Tokyo, Japan).

### 3.3. Preparation of Stachys *sp.* Extracts

Ten grams of each powdered plant material was extracted with 100 mL of 70% ethanol using ultrasound (Polsonic Palczyski Sp. J., Sonic 3, Warsaw, Poland) at 60 °C, for 30 min. The ethanolic extracts were filtered through paper filters into 100 mL flasks and then centrifuged (at 4500 rpm, 10 min) and the supernatant solutions were collected [29,49].

### 3.4. LC-MS/MS Analysis of Polyphenols

The study of flavonoids, as well as of the phenolic acids present in the six extracts of *Stachys* species was performed using the LC-MS/MS analysis on an Agilent 1100 HPLC Series system (Agilent, Santa Clara, CA, USA) equipped with: degasser (G1322A), binary gradient pump (G13311A), autosampler (G1313A), column thermostat, and UV detector (G1316A) that was coupled with an Agilent Ion Trap 1100 SL mass spectrometer (LC/MSD Ion Trap VL), according to the method previously published [24,50]. The standards used were from the class of phenolic acids (caftaric, gentisic, caffeic, chlorogenic, p-coumaric, ferulic, synapic acids), but also from the class of flavonoids (rutin, hyperoside, isoquercitrin, quercitrin, quercetin, myricetol, fisetin, patuletin, luteolin, kaempferol, apigen). The following parameters were used to separate the polyphenolic principles from plant extracts: a reversed-phase HPLC columns (Zorbax Agilent, Santa Clara, CA, USA, SB-C18, 100 mm × 3.0 mm id, 3.5 µm), mobile phase with methanol: 0.1% acetic acid (*v*/*v*), and a binary solvent gradient; the detection was performed using UV and MS with an electrospray ion source in negative mode. Other HPLC parameters: flow rate = 1 mL/min, injection volume = 5 µL, and column temperature = 48 °C with combined detection: UV (330 nm, 370 nm) and MS mode. The quantitative results were processed using the Agilent ChemStation software B01.03 and DataAnalysis version 5.3. 

Next, another previously described LC-MS method was used to determine 6 other polyphenols: epicatechin, catechin, syringic, gallic, protocatechuic, and vanillic acids [51]. The separation of the phenolic components was performed under the same chromatographic conditions mentioned above but with a different binary gradient, starting with 3% methanol and increasing to 8% in 3 min, 20% methanol for 8.5 min to 10 min, and finally, 3% methanol to re-equilibrate the column. An electrospray ion source operating in negative mode was used by the MS system in both analyses.

The polyphenolic substances in the *Stachys* extracts were identified by comparing their retention times and recorded electrospray mass spectra with those of the standards under the same conditions. Subsequently, the phenolic compounds were measured using their peak area and the matching standard’s calibration curve. The results were calculated in µg polyphenol/g dry weight (dw) of vegetal product.

### 3.5. LC-MS/MS Analysis of Rosmarinis Acid

Rosmarinic acid (RA) was quantified by the LC-MS/MS method described by Prof. L. Vlase [24]. An Agilent 1100 HPLC Series system (Agilent, Santa Clara, CA, USA) equipped as in Section 3.4 was used. The mobile phase used was different and prepared with acetonitrile and ammonium acetate in water (1 mM), the gradient elution: start with 5% acetonitrile and 3.3 min 25% acetonitrile. The flow rate was 1 mL/min. The autosampler injection volume was set at 25 μL. The results obtained were expressed as µg RA/g dry weight (dw) of vegetal product.

### 3.6. Total Polyphenols Content

Spectrophotometry is one of the most used methods for quantifying phenolic compounds in medicinal plants. The total polyphenol (TPC) contents of the seven Stachys extracts were measured by the Folin−Ciocâlteu method, according to the European Pharmacopoeia [52]. A similar method has been described previously [53]. Brief method: the extracts were treated with the Folin−Ciocâlteu reagent, and after dilution with distilled water, with a solution of sodium carbonate (290 g/L). After 30 min, the absorbance was measured at 760 nm. After performing the gallic acid calibration curve (R^2^ = 0.999), the results were calculated and expressed as mg of gallic acid equivalent (GAE)/mL plant extract [26,52]. Absorbance readings were taken in triplicates.

### 3.7. Flavonoid Content

The total flavonoid contents were spectrophotometrically determined, using a method previously described by us and based on the color reaction of the flavonoids with the aluminum chloride reagent (25 g/L). The absorbance of the yellow solution was measured at 430 nm [29]. Using a calibration curve (R^2^ = 0.999), the results were presented as mg of rutin equivalents (RE)/mL plant extract. Absorbance readings were taken in triplicates.

### 3.8. Caffeic Acid Derivative Content

The quantification of caffeic acid derivatives can be evaluated by colorimetric method with Arnow’s reagent (10.0 g sodium nitrite and 10.0 g sodium molybdate), according to the rules of the Romanian Pharmacopoeia X edition [31]. Absorbance was measured at 500 nm with the values of phenolic acids content of the *Stachys* extracts, expressed as caffeic acid equivalent (mg CAE/mL plant extract), using an equation derived from the calibration curve of caffeic acid (R^2^ = 0.994). Absorbance readings were taken in triplicates [29,53].

### 3.9. Antioxidant Activity

The antioxidant activity of the six *Stachys* extracts was examined by several methods: the DPPH radical scavenging method, the FRAP test (the ferric-reducing ability of plasma), nitrite-induced autooxidation of hemoglobin (NHA), inhibition of cytochrome *c*-catalyzed lipid peroxidation, and an electron paramagnetic resonance (EPR) spectroscopy assay [9,54,55].

#### 3.9.1. DPPH Radical Scavenging Assay

The antioxidant potentials of the *Stachys* sp. extracts were evaluated according to the DPPH method previously described by the authors [56,57]. This method measures the ability to remove stable free radicals DPPH by the natural antioxidant compounds (e.g., polyphenols) from plant extracts. A stock solution of 0.10 g/L DPPH in methanol (purple color) was prepared and 2.0 mL of this solution was added to 2.0 mL of extract solution (or standard) in methanol at different concentrations (12.50–250 μg/mL; yellow color). The absorbance of the *Stachys* extracts (As) and the control solutions (Ac = absorbance of DPPH with methanol, containing all reagents except the extracts) were spectrophotometrically measured at 517 nm, after 30 min (three replicates per treatment). Trolox was used as an antioxidant reference. The total antioxidant activity of the six extracts was expressed as IC_50_ (μg/mL): the concentration of plant material required to cause a 50% DPPH inhibition. The % of inhibition was plotted against concentration, and from the graphs, IC_50_ (R^2^: 0.999, 0.997, 0.996, 0.998, 0.999, 0.997) was calculated.

#### 3.9.2. FRAP Assay

The antioxidant capacity of the *Stachys* extracts was measured using the FRAP method [54,56,58]. After the preparation and treatment of the FRAP reagent with the samples, the absorbance of the obtained solutions was measured at 450 nm. The results were calculated in μmol Trolox Equivalent/mL *Stachys* sample.

#### 3.9.3. Nitrite-Induced Autooxidation of Hemoglobin

Hemoglobin and nitrite’s reaction was measured at 540 nm in a 50 mM pH 7 phosphate buffer. A total of 166 µM nitrite and 40 µM oxyhemoglobin were combined together with the samples. Origin 8 was utilized to calculate the inflection time (ti). The results are given in mg CAE/g (chlorogenic acid) plant for *Statcys* extracts. The linearity of the calibration curve is very good for chlorogenic acid (R^2^ = 0.99) as well as for catechin (R^2^ = 0.98) [59].

#### 3.9.4. Inhibition of Lipid Peroxidation Catalyzed by Cytochrome *c*

By sonication of lecithin (5.0 mg/mL) solubilized in 10 mM phosphate and at neutral pH, liposomes were produced. They were mixed with cytochrome *c* (2.0 µM) and *Stachys* extract (8.3 µg/mL). The reaction was monitored at 235 nm [60].

#### 3.9.5. Free Radical Generation Experiment

For the EPR experiment, the extracts were diluted at 0.05% and then mixed with 5 mM NaOH, in EtOH—90%. With a Bruker ELEXSYS E580 spectrometer (Bruker BioSpin GmbH, Rheinstetten, Germany) operating at room temperature and recording continuous waves at the X band, the EPR spectra were obtained [60].

### 3.10. Determination of Antimicrobial Activity

#### 3.10.1. Agar-Well Diffusion Method

The *Stachys* ethanolic extracts were evaluated for in vitro antibacterial and antifungal activities using the agar-well diffusion method [42]. These extracts were tested against five reference strains of microorganisms including two Gram-positive (*S. aureus*, *L. monocytogenes*), two Gram-negative (*S. enteritidis*, *E. coli*) bacteria and one yeast (*C. albicans*). Mueller–Hinton (MH) and Sabouraud dextrose (SD) broth and agar were used for bacteria and *C. albicans*, respectively. The plates containing specific agar were inoculated with a standardized inoculum prepared from pure colonies that were suspended in sterile broth to obtain a turbidity equivalent to 0.5 McFarland (1.0 × 10^6^ CFU/mL) standard (bio-Meriuex, Marcy l’Etoile, France). Six-millimeter diameter wells were cut from the inoculated agar plates using a sterile cork borer to allow the addition of tested extracts (50 μL) (three wells for each extract). After incubation at 37 °C for 24 h for bacteria and 48 h for *C. albicans*, the growth inhibition zones diameters were measured. Tests were conducted in triplicate, and clear halos greater than 10 mm were considered positive results. Gentamicin (10 µg) and fluconazole (25 µg) commercial disks were included as positive controls (standard antibiotics and antifungals agents). The negative control was 70% methanol with a diameter = 6 mm.

#### 3.10.2. Broth Microdilution Method

The in vitro antimicrobial efficacy was further investigated in terms of minimum inhibitory (MIC), bactericidal (MBC), and fungicidal (MFC) concentrations performing the broth microdilution method [45,58,61]. To determine these concentrations, each *Stachys* sp. extract was subjected to twofold serial dilutions using 100 µL of specific broth (MH and SD for bacteria and *C. albicans*, respectively). Each dilution was placed in two wells of sterile flat-bottomed 96-well microtiter plates (Deltalab, Barcelona, Spain) and incubated with a volume of 5.0 µL microorganism inoculum for 24 h at 37 °C. At the end of the incubation period, the wells were visually examined compared to the controls (the two types of broths used to culture bacterial and fungal species (MH and SD, respectively). No turbidity indicated an inhibitory effect, with the highest dilution associated with it determined as the MIC value. Next, a volume of 10.0 µL from each well was cultured on the corresponding agar plates. No visible growth on these plates after 24 h and 48 h for bacteria and *C. albicans*, respectively, pointed out the MBC and MFC values. This assay included controls such as gentamicin 50 mg/mL (Sigma-Aldrich, St. Louis, MO, USA), and fluconazole (10–1000 μM) (Sigma-Aldrich, St. Louis, MO, USA) and was performed in duplicates for each tested product.

#### 3.10.3. Anti-Biofilm Assay

The inhibitory activity of *Stachys* sp. extracts against two stages of biofilm formation, biofilm attachment (T0) and destruction of 24 h pre-formed biofilm (T24), respectively, was investigated using previously reported protocols [45,61,62]. The bacterial and fungal inoculums were prepared as presented for the disk diffusion; for T0 the inoculums were placed in sterile flat-bottomed 96-well microtiter plates to be incubated with equal volumes (100 μL) of each *Stachys* extract for 24 h at 37 °C without shaking. In the case of T24, each inoculum was initially cultured for 24 h to obtain the biofilm and further treated with equal volumes of each *Stachys* extract. Controls such as organisms + specific broth, bacteria + MH broth + gentamicin, *C. albicans* + SD broth + fluconazole were included. Following 24 h incubation, the biofilm biomass was quantified using the crystal violet staining (CVS) assay. The content of each well was removed and the 96-well microtiter plates were washed three times using sterile distilled water, a step required to remove the unattached microorganism cells. The plates were dried and added with 96% methanol (150 μL) for 20 min and the fixed adhered cells were stained with 100 μL of 0.1% crystal violet solution (Sigma-Aldrich, St. Louis, MO, USA) for 20 min at room temperature. The plates were washed five times with sterile distillated water. The crystal violet stain bound to the adherent cells of each well was re-solubilized with 150 μL of 100% ethanol. After gentle shaking, the plates’ absorbance (optic density, OD) was read at 490 nm using a microplate reader Sunrise™ (Tecan, Männedorf, Switzerland). and the results were expressed as percentage inhibition using the equation below [45]. 

Inhibition (%) = (ODcontrol − ODExtract)/ODcontrol × 100. Based on the inhibition (%) calculated values, the tested extract activity was described as good (above 50%, ++), poor (0–50%, +), and no inhibition, or enhancement of biofilm development and growth (<0, -) [45].

### 3.11. Statistical Data Analysis

All experiments were performed in triplicate or more and all data were expressed as mean ± standard deviation using the Excel software package 2016. The statistical analysis of differences was performed using one-way analysis of variance (ANOVA), with *p* < 0.05 as the threshold value for statistical significance: very significant (*p* < 0.001), significant (0.001 < *p* < 0.05), and insignificant (*p* > 0.05).

## 4. Conclusions

This is a detailed report on the phytochemical composition and antioxidant and antimicrobial properties of extracts obtained from six medicinal species of *Stachys* from Romania: *S. officinalis*, *S. germanica*, *S. byzantina*, *S. sylvatica*, *S. palustris*, and *S. recta*. Fifteen phenolic compounds were found in the six species using LC-MS, of which seven substances were common: protocatechuic acid, rosmarinic acid, gentisic acid, vanillic acid, *p*-coumaric acid, ferulic acid, luteolin, and apigenin. Chlorogenic acid was found in large amounts; the most abundant species were *S. germanica* (4205.2 μg/g) *S. sylvatica* (5552.7 μg/g), and *S. recta* (6761.4 μg/g). Furthermore, significant antioxidant properties were demonstrated using conventional and innovative methods for *S. byzantina*, *S. germanica* and *S. palustris*-derived extracts. These extracts also displayed the most intense in vitro antimicrobial potential. These results provide a scientific foundation and open important perspectives for developing phytopharmaceuticals indicated in various pathologies that involve antioxidant and antimicrobial mechanisms.

## Figures and Tables

**Table 1:** Phenolic compounds identified by LC-MS/MS in *Stachys* sp. extracts (μg/g dw).

Phenolic Compounds	*m*/*z* Value (Precursor)	*m*/*z* Value (Daughter Ions; Collision Energy)	tR ± SD (min)	*S*. *officinalis*	*S*. *germanica*	*S*. *byzantina*	*S*. *sylvatica*	*S*. *palustris*	*S*. *recta*
Gallic acid	169	169 (0.5 V)	1.50 ± 0.01	-	-	-	-	141.5 ± 8.4	-
Rosmarinic acid	359	160.7; 178.6; 196.7 (0.9 V)	2.20 ± 0.01	43.4 ± 0.5	7.8 ± 0.01	3.0 ± 0.02	1.9 ± 0.02	2.4 ± 0.01	2.6 ± 0.05
Protocatechuic acid	153	153 (0.5 V)	2.80 ± 0.01	94.6 ± 0.5	83.5 ± 0.4	28.6 ± 0.3	100.7 ± 9.2	94.2 ± 1.7	157.8 ± 2.1
Gentisic acid	153	108.7 (0.9 V)	3.52 ± 0.04	<0.2	<0.2	<0.2	<0.2	<0.2	<0.2
Caftaric acid	311	148.6; 178.6 (0.9 V)	3.54 ± 0.05	4.7 ± 0.2	-	-	-	-	-
Chlorogenic acid	353	178.7; 190.7 (0.9 V)	5.62 ± 0.05	1131.8 ± 15.8	4205.2 ± 44.7	2012.1 ± 37.8	5552.7 ± 47.2	3595.2 ± 24.7	6761.4 ± 58.5
Vanillic acid	167	167 (0.5 V)	6.70 ± 0.01	83.5 ± 0.7	21.1 ± 0.1	16.4 ± 0.3	16.6 ± 0.2	6.2 ± 0.08	20.3 ± 0.5
Syringic acid	197	197 (0.5 V)	8.40 ± 0.01	82.7 ± 0.2	-	7.0 ± 0.06	8.6 ± 0.1	4.4 ± 0.3	-
*p*-Coumaric acid	163	118.7 (0.9 V)	9.48 ± 0.08	27.8 ± 0.1	6.2 ± 0.08	4.4 ± 0.08	6.2 ± 0.3	14.0 ± 0.4	32.0 ± 0.9
Ferulic acid	193	133.7; 148.7; 177.6 (0.9 V)	12.80 ± 0.10	11.1 ± 0.4	5.0 ± 0.02	3.5 ± 0.04	3.0 ± 0.06	6.0 ± 0.2	<0.2
Isoquercitrin	463	254.9; 270.9; 300.7; 342.8 (1.2 V)	19.60 ± 0.1	18.9 ± 0.1	233.1 ± 6.8	-	86.2 ± 3.7	-	72.8 ± 2.1
Rutin	609	254.9; 270.9; 300.7; 342.8 (1.2 V)	20.20 ± 0.15	<0.2	-	-	-	-	-
Quercitrin	447	178.8; 300.7 (1.2 V)	23.64 ± 0.13	-	-	-	-	<0.2	-
Luteolin	285	150.6; 174.6; 198.6; 240.7 (1.5 V)	29.10 ± 0.19	15.7 ± 0.2	26.8 ± 0.1	12.2 ± 0.1	<0.2	3.9 ± 0.2	<0.2
Apigenin	269	148.6; 150.6; 224.7; 226.7 (1.5 V)	33.10 ± 0.15	84.2 ± 1.7	21.4 ± 0.2	27.3 ± 0.6	13.6 ± 0.2	<0.2	<0.2

“-” not found, below limit of detection. Values are the mean ± SD (*n* = 3).

**Table 2:** Polyphenolic contents of *Stachys* sp. extracts.

*Stachys* sp. Extracts	TPC (mg GAE/mL)	Flavonoids (mg RE/mL)	Caffeic Acid Derivatives (mg CAE/mL)
*S. officinalis* (SO)	2.51 ± 0.09 ^a^	1.18 ± 0.01 ^d^	0.20 ± 0.01 ^g^
*S. germanica* (SG)	5.98 ± 0.21 ^a^	3.30 ± 0.11 ^f^	0.88 ± 0.01 ^i^
*S. byzantina* (SB)	6.40 ± 0.11	3.90 ± 0.21	0.89 ± 0.05
*S. sylvatica* (SS)	4.00 ± 0.13 ^a^	1.22 ± 0.06 ^d,f^	0.20 ± 0.03 ^h^
*S. palustris* (SP)	5.35 ± 0.29 ^b,c^	3.14 ± 0.17 ^f^	0.72 ± 0.06 ^g,i^
*S. recta* (SR)	3.78 ± 0.17 ^a,c^	1.13 ± 0.09 ^e^	0.23 ± 0.08 ^h,i^

Each value is the mean ± SD of three independent measurements; GAE, RE, CAE: gallic acid, rutin, caffeic acid equivalents. Lowercase letters in the same row indicate significant differences (*p* < 0.05). ^a^ *p* < 0.001 (SB vs. SO, SG, SS, SR); ^b^ *p* < 0.05 (SB vs. SP); ^c^ *p* > 0.05 (SG vs. SP, SS vs. SR); ^d^ *p* < 0.001 (SB vs. SO, SS); ^e^ *p* < 0.05 (SB vs. SR; SG vs. SR); ^f^ *p* > 0.05 (SO vs. SS, SO vs. SP, SB vs. SP, SG); ^g^ *p* < 0.001 (SB vs. SO, SP); ^h^ *p* < 0.05 (SG vs. SS, SR; SB vs. SS, SR; SS vs. SR); ^i^ *p* > 0.05 (SO vs. SP, SR, SB vs. SG, SP vs. SR).

**Table 3:** Antioxidant activity of *Stachys* sp. extracts.

*Stachys* sp. Extracts	DPPH (IC_50_ µg/mL)	FRAP (μM TE/mL)	NHA mg CATE/g
*S. officinalis*	139.16 ± 3.80 ^a^	280.17 ± 1.83 ^b^	23.44 ± 3.24
*S. germanica*	54.18 ± 1.16 ^a^	688.21 ± 5.91	66.59 ± 5.37
*S. byzantina*	53.61 ± 1.19 ^a^	354.88 ± 3.11 ^b^	27.06 ± 3.00
*S. sylvatica*	76.04 ± 0.35 ^a^	218.01 ± 0.98 ^b^	20.01 ± 6.78
*S. palustris*	54.82 ± 1.33 ^a^	502.43 ± 5.56 ^b^	59.38 ± 2.54
*S. recta*	84.94 ± 1.45 ^a^	305.15 ± 2.84 ^b^	37.49 ± 1.96
Trolox	11.19 ± 0.09	-	-

Each value is the mean ± SD of three independent measurements; IC_50_: half-maximal inhibitory concentration; TE: Trolox equivalents; NHA: nitrite-induced hemoglobin autooxidation; CATE: Catechin equivalents. Lowercase letters in the same row indicate statistical differences: ^a^ *p* < 0.001 (Trolox vs. SO, SG, SB, SS, SP, SR); ^b^ *p* < 0.001 (SG vs. SO, SB, SS, SP, SR).

**Table 4:** In vitro antibacterial activity of *Stachys* sp. ethanolic extracts by agar-well diffusion method.

Inhibition Zone Diameter (IZD, mm)					
*Stachys* sp. Extracts	*Staphylococcus aureus*	*Listeria* *monocytogenes*	*Escherichia coli*	*Salmonella enteritidis*	*Candida albicans*
*S. officinalis*	16 ± 0.50 ^b^	14 ± 1.00 ^a^	10 ± 1.00 ^a^	10 ± 0.25 ^a^	12 ± 0.50 ^d^
*S. germanica*	20 ± 0.25 ^a^	18 ± 0.50 ^a^	8 ± 0.25 ^a^	10 ± 0.50 ^a^	10 ± 0.50 ^d^
*S. byzantina*	20 ± 0.25 ^a^	18 ± 0.00 ^a^	8 ± 0.50 ^a^	8 ± 0.50 ^a^	12 ± 1.00 ^d^
*S. sylvatica*	16 ± 0.75 ^b^	16 ± 0.25 ^a^	8 ± 1.00 ^a^	8 ± 1.00 ^a^	10 ± 0.50 ^d^
*S. palustris*	18 ± 0.50 ^c^	16 ± 1.00 ^a^	8 ± 0.00 ^a^	8 ± 0.25 ^a^	12 ± 0.50 ^d^
*S. recta*	16 ± 0.00 ^b^	16 ± 1.00 ^a^	10 ± 0.25 ^a^	12 ± 0.50 ^b^	12 ± 0.00 ^d^
Gentamicin	18 ± 0.25	22 ± 0.50	18 ± 0.25	19 ± 1.00	-
Fluconazole	-	-	-	-	21 ± 0.00

Values are means of triplicate determination (n = 3) ± standard deviations. Lowercase letters in the same row indicate significant differences (*p* < 0.05). ^a^ *p* < 0.001 (extracts vs. gentamicin); ^b^ *p* < 0.05 (*S. sylvatica*, *S. recta* vs. gentamicin); ^c^ *p* > 0.05 (*S. palustris* vs. gentamicin); ^d^ *p* < 0.001 (extracts vs. fluconazole); gentamicin (10 μg/disk) and fluconazole (25 μg/disk) were used as positive controls.

**Table 5:** In vitro antibacterial activity of *Stachys* sp. ethanolic extracts using the broth microdilution method.

*Stachys* sp. Extracts	*Staphylococcus aureus*		*Listeria monocytogenes*		*Escherichia coli*		*Salmonella* *enteriditis*		*Candida albicans*	
	MIC	MBC	MIC	MBC	MIC	MBC	MIC	MBC	MIC	MFC
*S. officinalis*	0.705	1.411	1.411	1.411	1.411	1.411	1.411	1.411	0.352	1.411
*S. germanica*	0.423	0.423	0.847	1.695	3.39	3.39	3.39	3.39	1.695	3.39
*S. byzantina*	0.468	0.468	1.875	3.75	3.75	3.75	3.75	3.75	0.937	3.75
*S. sylvatica*	1.20	2.40	1.20	2.40	2.40	2.40	2.40	2.40	1.20	2.40
*S. palustris*	0.817	1.635	1.635	3.27	3.27	3.27	3.27	3.27	0.817	3.27
*S. recta*	1.12	2.24	1.12	2.24	2.24	2.24	2.24	2.24	0.56	2.24
Gentamicin MIC (mg/L)	3		4		3		3		-	
Fluconazole MIC (mg/L)	-		-		-		-		8	

“-” = not active; MIC: minimum inhibitory concentration (μmol GAE/mL); MBC: minimum bactericidal concentration (μmol GAE/mL); MFC: minimum fungicidal concentration (μmol GAE/mL).

**Table 6:** Antibiofilm activity of *Stachys* sp. ethanolic extracts.

			% Inhibition							
*Stachys* sp. Extracts	*Staphylococcus aureus*		*Listeria monocytogenes*		*Escherichia* *coli*		*Salmonella* *enteriditis*		*Candida albicans*	
	T0	T24	T0	T24	T0	T24	T0	T24	T0	T24
*S. officinalis*	+	-	+	-	+	-	-	+	++	+
*S. germanica*	++	++	++	+	+	-	-	+	+	+
*S. byzantina*	++	++	+	+	+	-	-	++	++	+
*S. sylvatica*	++	+	+	+	++	+	-	-	++	-
*S. palustris*	++	+	++	+	+	-	-	+	++	-
*S. recta*	+	+	+	-	+	-	-	-	++	-
Gentamicin	+	++	+	++	-	++	-	++	-	-
Fluconazole	-	-	-	-	-	-	-	-	++	++

The extracts antibiofilm activity was described based on the inhibition (%) calculated values, as good (above 50%, ++), poor (0–50%, +), and no inhibition, or enhancement of biofilm development and growth (<0, -) [45].

## Data Availability

Data are contained within the article.
